# Turkish Propolis and Its Nano Form Can Ameliorate the Side Effects of Cisplatin, Which Is a Widely Used Drug in the Treatment of Cancer

**DOI:** 10.3390/plants9091075

**Published:** 2020-08-21

**Authors:** Pinar Tatli Seven, Ismail Seven, Selcan Karakus, Seda Iflazoglu Mutlu, Gozde Arkali, Yesim Muge Sahin, Ayben Kilislioglu

**Affiliations:** 1 Department of Animal Nutrition and Nutritional Diseases, Firat State University, 23119 Elazig, Turkey; ptatli@firat.edu.tr (P.T.S.); siflazoglu@firat.edu.tr (S.I.M.); 2 Department of Plant and Animal Production, Firat State University, 23119 Elazig, Turkey; 3 Department of ChemistryIstanbul University-Cerrahpasa, 34320 Istanbul, Turkey; selcan@istanbul.edu.tr (S.K.); ayben@istanbul.edu.tr (A.K.); 4 Department of Physiology, Firat State University, 23119 Elazig, Turkey; garkali@firat.edu.tr; 5 Department of Biomedical Engineering, Istanbul Arel University, 34320 Istanbul, Turkey; ymugesahin@arel.edu.tr

**Keywords:** propolis, nano-propolis, cancer, cisplatin, oxidative damage, apoptosis, rats

## Abstract

This study was performed to determine the effects of chitosan-coated nano-propolis (NP), which is synthesized via a green sonochemical method, and propolis on the side effects of cisplatin (CP), which is a widely used drug in the treatment of cancer. For this aim, 56 rats were divided into seven groups, balancing their body weights (BW). The study was designed as Control, CP (3 mg/kg BW at single dose of CP as intraperitoneal, ip), Propolis (100 mg/kg BW per day of propolis by gavage), NP-10 (10 mg/kg BW of NP per day by gavage), CP + Propolis (3 mg/kg BW of CP and 100 mg/kg BW of propolis), CP + NP-10 (3 mg/kg CP and 10 mg/kg BW of NP), and CP + NP-30 (3 mg/kg BW of CP and 30 mg/kg BW of NP). Propolis and NP (especially NP-30) were preserved via biochemical parameters, oxidative stress, and activation of apoptotic pathways (anti-apoptotic protein: Bcl-2 and pro-apoptotic protein: Bax) in liver and kidney tissues in the toxicity induced by CP. The NP were more effective than propolis at a dose of 30 mg/kg BW and had the potential to ameliorate CP’s negative effects while overcoming serious side effects such as liver and kidney damage.

## 1. Introduction

Cancer is one of the most prominent health problems of our age and, although it used to be ranked as seventh and eighth among terminal illnesses in the early part of the century, today it is ranked as second, following cardiac diseases in many countries of the world including Turkey. Treatment methods of cancer usually include chemotherapy, radiotherapy, surgery, and immunotherapy and one or several of these methods is/are used in the treatment according to individual characteristics of patients diagnosed with cancer and the severity of illness. Chemotherapy is a treatment method that has selective lethal effects, especially against multiplying cells, and is performed with natural or synthetic chemical and biological agents, and hormones. The aim of chemotherapy is to extend patients’ life expectancy and provide a better quality of life. However, there are chemotherapy-related difficulties and toxic effects depending on the method used [1]. Antioxidant substances have been commonly used in recent studies in order to enhance the immune system and reduce cytotoxic effects [2]. Hazardous chemicals’ wastes containing cisplatin (CP) are released into the environment in various departments of hospital laboratories and engineering departments. In addition, the chemical waste of anticancer agents of industrial and healthcare institutions can pose a vital risk to nontarget species in the aquatic environment due to cytotoxic, genotoxic, mutagenic, and teratogenic effects. When environmental problems are considered, nonhazardous chemicals in nano-technological products gain importance for the continuation of human and animal health. The common types of nonhazardous and natural chemicals, such as propolis, clay, sugar, etc., are used in the nano-formulations. However, it is reported that nanotechnology-applied products might be more effective than conventional products [3]. With the recent advances in nanotechnological studies, nanomaterials are widely preferred for several industrial and pharmaceutical applications [4,5].

Nanotechnological effect is provided through using materials (such as chitosan) that inhibit enzyme activation and DNA synthesis [6]. The CP is one of the most commonly used chemotherapeutic drugs and a platinum-based chemotherapy substance making the bacteria-DNA synthesis impossible and, thus, leading to the death of cells that cannot repair the DNA. It causes a number of cytotoxic side effects while producing these effects. The side effects of its clinical use were primarily reported as nephrotoxicity and hepatotoxicity [2,7]. The increase in liver enzymes in serum and bilirubin is an indicator of liver dysfunction [8]. CP hepatotoxicity indicates that the cytochrome P450-2E1 enzyme worsens toxicity even further with the increase in expression level [9]. Histopathological changes are sinus dilatation and infiltration of inflammatory cells around the portal area, as well as necrosis and degeneration of hepatocytes [10,11]. These conditions, caused by oxidative stress, may lead to even worse situations. As a matter of fact, reactive oxygen species (ROS) causing the development of oxidative stress may induce apoptosis by means of the intrinsic and extrinsic pathways [12]. In the extrinsic pathway of apoptosis, ROS are formed by the Fas ligand as an upward stream for the activation of Fas by phosphorylation. This condition is necessary not only for the induction of apoptosis, but also for the post-mortality domain related with caspase 8 and Fas [13]. While the ROS in the intrinsic pathway facilitate the release of cytochrome C by activating Bcl-2 and Bcl-xL, which are known pore-stabilizing proteins, they inhibit the proteins that are pore-destabilizing (Bcl-2 associated protein X, death receptor signal Bcl-2) [14]. Indeed, high levels of ROS and CP may cause apoptosis and necrosis in cells with cancer [15,16,17]. ROS may also induce necrosis via autophagia [18].

The term nanotechnology was used for the first time to define the ultra-fine production technology in 1974 [19,20]. Nanotechnology provides progress in molecular, atomic, and macromolecular areas [20]. The purpose of nanotechnology is to increase the effectiveness of materials by using the change in their size and to enable nanosize to result in a better effect in the fields of biology and medicine [21]. As a matter of fact, when active ingredients are present in the nano structure, they increase the stability of substances due to their protective effects on oxidant agents and other components or enzymes [20,22,23]. In recent years, polymeric nanoparticles with unique properties have been preferred for use in different industrial applications. The use of biodegradable and biocompatible polymer matrix-based nanoparticles released into soil, water, and air without pollution plays an important role in reducing the environmental burden. Plastic waste is a major environmental concern, so biodegradable polymer matrix-based nanoparticles with a small size (1 to 100 nanometers) and a high surface area are an environmentally friendly alternative to conventional plastic. However, biodegradable polymeric nanoparticles have played a significant role in recent research, as they undergo complete degradation and have a less negative impact on the environment [24,25]. The presence of biopolymers has been important for the environment and nanotechnology to ensure the uniform distribution of nanoparticles, prevent their aggregation, and increase stability. For this purpose, chitosan, a green material, was preferred as a biopolymer matrix to prevent the formation of secondary pollutants [26]. Chitosan is a biocompatible polymer for interaction of the material and the body and it is also used for the interaction between material and body due to the presence of free amino groups, which favor the interaction with cells. It can be chemically modified to prepare the nanocarrier, which is controlled and adsorbed in the body [27,28].

Propolis, which is a natural beekeeping product, due to the beneficial effects of its compounds, has been used for a lot of pharmacological and biological activities [29]. When administered orally, propolis is nontoxic and rarely causes allergic reactions. The allergic reactions of propolis are more common after topical administration. Propolis is considered an occupational contact allergen for 0.76–4.3% of beekeepers [30,31]. Nanoscale encapsulation of propolis with chitosan was conducted in the present study. There are studies in the literature indicating that the nanoscale drugs have little allergic effects [32,33] or side effects [34]. Moreover, supporting studies also take part in the literature suggesting that chitosan encapsulation ameliorates hypersensitivity [33,34,35,36]. Biomaterial-based nano-propolis (NP) can be synthesized by different techniques such as hydrothermal, sonochemical, microwave, and solvothermal [37,38,39,40]. There are several studies on biomaterial-based NP in different applications, such as biomedical and purification of wastewater, and relatively few studies on the preparation of propolis-based NP.

In this study, propolis-based nanoparticles, which are a natural material with a homogeneous distribution in the polymer matrix, were synthesized by the green sonochemical method. The low cost and nontoxic, natural materials attract the attention of nanotechnology studies. For this reason, the propolis is a green candidate for these new nanomaterials. Having many important effects, propolis dissolves in water slightly. On the other hand, NP may prove more effective by increasing the dissolvability of propolis. Studies on NP usually remain limited to studies on bacteria. As a matter of fact, in studies on *E. coli,* it has been reported that even a very small amount of NP inhibits the development of *E. coli* [41,42]. It was reported that antimicrobial activity of NP was much more effective than propolis [21]. In addition, antitumor studies have revealed that the antiproliferative effects of NP are much better than propolis [3]. The hypoglycemic effect of NP has been investigated, as well. It was concluded that NP, used in the treatment of diabetic rats induced by streptozotocin, is effective in the renewal of damaged β-cells and reduction in blood glucose (GLU) level [43]. In the light of all these studies, the basis of our hypothesis was based on the assumption that the nanoparticle structure of propolis, which is known to be effective on oxidative damage, could be more effective. Studies are needed to confirm the effectiveness of different doses of NP in different types of cancer in order to reduce the effects of the damage caused by CP, which is one of the most common drugs used in the treatment of cancer that significantly affects human health.

This study was designed for the purpose of determining the effects of NP in rats induced by CP on performance (FI, BW and BWC) and some biochemical parameters and, also antioxidant status and Bcl-2 and Bax protein expression levels in liver and kidney tissues.

## 2. Results

### 2.1. Body Weight, Body Weight Change and Feed Intake

In the present study, body weights (BW) (*p* < 0.01) on day 21, BWC (*p* < 0.01) and FI (*p* < 0.05) in the 1–21 day period of rats treated with CP administration were significantly decreased in comparison with those of the control (Table 1). CP + Propolis administration caused a significant increase in BWC in the 1–21 day period, and FI between day 15 and 21, when compared to the CP-only group (*p* < 0.01).

### 2.2. Biochemical Parameters

Biochemical parameters of the study groups are given in Table 2. The biochemical parameters were affected by CP administration. CP treatment caused a significant increase in GLU (*p* < 0.001), aspartate aminotransferase (AST) (*p* < 0.01), alanine aminotransferase (ALT) (*p* < 0.05), alkaline phosphatase (ALP) (*p* < 0.001), creatinine (*p* < 0.05) and blood urea nitrogen (BUN) (*p* < 0.001) levels when compared with the control group (Table 2). CP + Propolis and CP + NP administrations caused a significant difference in AST (*p* < 0.01), GLU, ALP, albumin and BUN (*p* < 0.001) levels when compared with the CP-only group.

### 2.3. Antioxidant Status in Liver and Kidney Tissues

CP treatment caused a significant increase in MDA level and significant decreases in GSH level, GSH-Px and CAT activity when compared with the control group in the liver and kidney tissues (*p* < 0.001; Table 3). Administration of propolis (CP+propolis) and NP(CP+NP) together with CP-treatment significantly decreased the MDA level and significantly increased the GSH level, GSH-Px and CAT activity when compared with the CP-only group (*p* < 0.001; Table 3). Interestingly, MDA activity of CP + NP groups were found similar to control group in liver and kidney tissues, and GSH level and GSH-Px activity of the CP + NP-30 group were found higher than the control group in liver tissue (*p* < 0.001). Besides, GSH level and CAT activity in kidney tissues of the CP + NP-30 group were found higher than in the control group (*p* < 0.001). Interestingly, MDA, GSH levels and GSH-Px activity of the CP + NP-30 group were found similar to the control group, and CAT activity in the same group was found higher than in the control group (*p* < 0.001).

### 2.4. Bcl-2, Bax Protein Expression of Liver and Kidney Tissue

**Figure 1:**
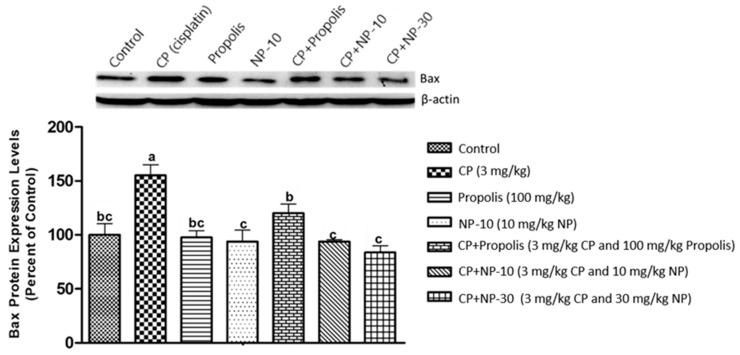
Bax protein expression levels in liver tissue. a,b,c: Mean values within a row differ significantly, Significance is *p* < 0.001 (NP: nano-propolis).

**Figure 2:**
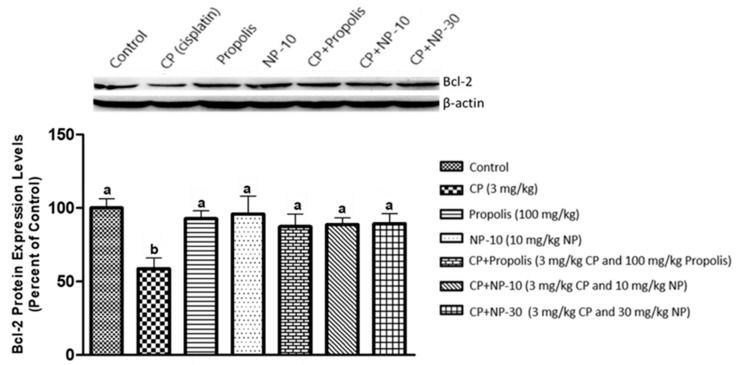
Bcl-2 protein expression levels in liver tissue. a,b,c: Mean values within a row differ significantly, Significance is *p* < 0.001 (NP: nano-propolis).

**Figure 3:**
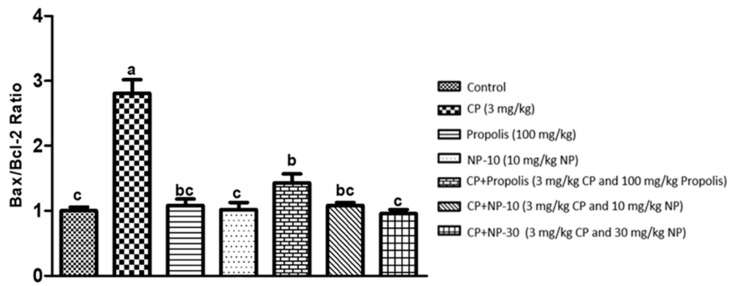
Bax/Bcl-2 protein expression ratio in liver tissue. a,b,c: Mean values within a row differ significantly, Significance is *p* < 0.001 (CP: cisplatin; NP: nano-propolis).

**Figure 4:**
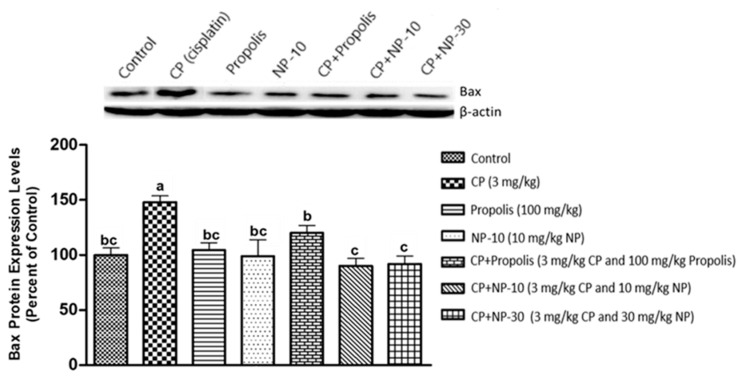
Bax protein expression levels in kidney tissue. a,b,c: Mean values within a row differ significantly, Significance is *p* < 0.001 (NP: nano-propolis).

**Figure 5:**
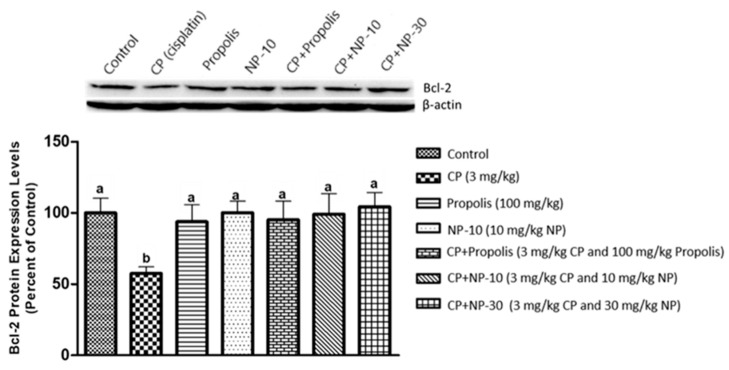
Bcl-2 protein expression levels in kidney tissue. a,b,c: Mean values within a row differ significantly, Significance is *p* < 0.001 (NP: nano-propolis).

**Figure 6:**
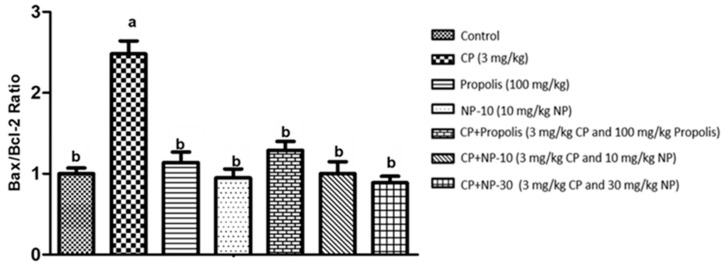
Bax/Bcl-2 protein expression ratio in kidney tissue. a,b,c: Mean values within a row differ significantly, Significance is *p* < 0.001 (CP: cisplatin; NP: nano-propolis).

The apoptotic process was evaluated by detecting the expressions of apoptotic markers i.e., Bax and Bcl-2 in liver and kidney tissue of all groups (Figure 1, Figure 2, Figure 3, Figure 4, Figure 5 and Figure 6). Respectively, Figure 1, Figure 2, Figure 4 and Figure 5 show the Western blot bands of the expression levels of Bcl-2 and Bax in the liver and kidney tissues of all groups. CP administration increased the expression of pro-apoptotic protein Bax and decreased the expression of anti-apoptotic protein Bcl-2 in liver and kidney tissues (Figure 1, Figure 2, Figure 4 and Figure 5) when compared to the control group (*p* < 0.001). In contrast, treatments with propolis and NP attenuated apoptosis in liver and kidney tissues (*p* < 0.001). In addition, Bax and Bcl-2 proteins’ expression ratios in liver and kidney tissues are shown in Figure 3 and Figure 6. The highest Bax/Bcl2 ratio in both tissues were determined in the CP-only group (Figure 3 and Figure 6) (*p* < 0.001).

## 3. Discussion

CP is one of the most important chemotherapeutic agents. However, side effects of CP restrict its usage and effectiveness. Hepatotoxicity, nephrotoxicity, decrease in sperm, hair loss, nausea and vomiting are some important side effects of it. The studies have usually suggested that CP has a toxic effect with a mechanism caused by oxidative damage. It has been reported that the CP causes the production of free oxygen radicals and nuclear Factor kappa B activation and increases the adenosine A1 receptor synthesis [44]. In addition, it is reported that CP inhibits the DNA transcription by making cruciate ligaments in the chain. The effect of CP on nephrotoxicity can be explained with the fact that inosine, which is an adenosine metabolite, creates an oxidative damage leading to the production of free radicals while being metabolized by xanthine oxidase [44,45].

In this study, the effect on FI of CP was investigated and it was determined that the FI was statistically reduced by CP (Table 1). Similar to the present study; previous studies on CP reported that FI was significantly reduced by CP in rats [46]. It was reported that CP has direct toxic effects on the stomach [46]. Similarly, the study conducted by Malik et al. [46] found that FI was significantly reduced by CP 6 mg/kg BW in rats. They associated this effect of CP with the fact that it causes gas accumulation in the stomach and emptying of the stomach was delayed due to this accumulation of gas. It was seen that propolis and NP applications positively affected the decrease in FI caused by CP toxicity, and FI in the CP + Propolis and CP + NP-30 groups was similar to the control group between days 1 and 21. When examining BW and BWC, it was determined that the CP group had significantly lower values than the control group between days 1 and 21. When examining the values of the CP + Propolis group between days 1 and 21, it was determined that there was a significant increase compared to the CP group. It was observed that although there was an improvement in FI and an increase in BW in the CP + NP groups compared to the CP group, there was a more significant improvement in the CP + Propolis group. This was associated with the fact that propolis increased FI and especially resin, wax, honey and vanillin in its structure [47,48] caused the flavour increase. In addition, the presence of significant differences between propolis doses of nano groups (10 and 30 mg/kg BW) and the propolis group (100 mg/kg BW) was associated with the fact that flavour-increasing substances were partially fewer than the nano groups. Improvements in FI reflect on BW values, as well. Additionally, improvements in FI and BW were associated with flavones in the structure of propolis [49].

When examining biochemical parameters in the present study, it was determined that GLU, AST, ALP, albumin and BUN values were statistically different in the CP group. However, the CP + Propolis and CP + NP application significantly changed this difference in a positive way (Table 2). In a study conducted on rats with liver damage induced by CP, Cetin et al. [50] investigated the effects of grape seed and essential thyme oil on some serum blood parameters. They determined that CP caused a statistically significant increase in serum ALT and AST levels. The most important characteristics of the curative substances is that they have antioxidant compounds (especially phenolic compounds) as in propolis. In a previous study conducted with propolis, it was reported that ALP and AST levels of male rats in which oxidative stress was induced with alcohol caused a significant decrease in the group in which propolis was applied [51]. The effect of CP is observed not only to increase in liver enzymes, but also on renal toxicity. When examining creatinine and BUN values in the present study, it was found that creatinine and BUN values of the CP group were significantly higher than other groups; whereas, BUN values of the CP + Propolis and CP + NP groups were significantly lower than the CP group. In the study conducted by Katanic et al. [52] using spirea (Filipendula Ulmaria) extract to eliminate the side effects of CP, it was reported that CP significantly increased ALT, AST, and ALP values, which are among liver function tests, and creatinine and BUN values, which are among renal function tests, compared to the control group; on the other hand, the aforementioned extract (derived from the root area) decreased these parameters compared to the group to which CP was administered alone, but did not decrease the total protein, which is similar to the present study. The fact that propolis and NP had therapeutic effects against the negative effects of CP on blood parameters was associated with effective antioxidant compounds in their structure [53,54].

Antioxidant and anticancer effects of propolis have become the subject of recent studies [55,56]. In studies conducted with propolis and other antioxidant substances, it has been reported that propolis reduces the formation of free radicals and lipid peroxidation by means of flavonoids, which are available in structure of it [55,57]. Flavonoids display an antioxidant property by chelating with trace elements or radicals [58]. It is reported that flavonoids protect unsaturated fatty acids against oxidants in cell membranes, just like ascorbates [59]. Caffeic acid phenethyl ester (CAPE), one of the main ingredients of propolis, blocks the production of ROS [60]. The results of previous studies reported that CP caused damage in the liver tissue by increasing the MDA level and decreasing antioxidant enzymes; however, antioxidant contributions minimized that damage [50,61]. In a study using grape seed extract to determine whether or not especially effective phenolic compounds could reduce liver damage [50], it was found that superoxide dismutase and GSPx activities in the liver tissue were significantly higher in the CP + grape seed extract group compared to the CP alone. In the present study, similar results were obtained in the liver tissue (Table 3). As a matter of fact, when examining the MDA level in the liver and kidney tissues, it was determined that the CP group was significantly higher than the other groups; whereas, GSH, GSH-Px and CAT levels decreased. It was found that the MDA level of the liver and kidney tissues of the CP + Propolis group significantly decreased compared to the CP group; whereas, GSH, GSH-Px and CAT levels increased significantly in the liver and kidneys (Table 3). It is reported that various flavonoids and phenolic substances in propolis act like vitamin C and prevent lipids and other compounds from oxidating or they have the ability of scavenging free radicals by interrupting the oxidative damage process [51,57]. In addition, flavonoids inhibit the activity of enzyme systems including lipid peroxidation, thrombocyte aggregation, capillary permeability, fragility, cyclooxygenase and lipoxygenase [59]. In parallel with the results of previous studies [51,57], the results of this study showed that propolis reduced oxidative damage caused by CP toxicity in tissues. In the study, propolis was used in the level of 100 mg/kg BW and the results were found to be effective on the antioxidant system. On the other hand, the effectiveness of NP, which was used at lower doses (10 and 30 mg/kg BW) than propolis (100 mg/kg BW) was compared with propolis. In the obtained results, it was primarily determined that CP + NP groups, similarly with the propolis group, reduced MDA levels in all tissues compared to the CP group and both CP + NP groups significantly increased GSH, GSH-Px and CAT levels in all tissues. It was found that liver and kidney MDA levels in the CP + NP groups were similar to the control group and GSH, GSH-Px and kidney CAT levels in the liver and kidney tissues also had similar results to the control group. These results showed that both doses of the NP application were effective on the recovery of oxidative damage caused by CP in tissues and it was found that especially the NP dose of 30 mg/kg BW was relatively more effective. In previous studies, the effect of nano-selenium on testicle tissues, in which oxidative damage was induced with CP, was examined and it was determined that nano-selenium increased antioxidant enzyme activities in testicle tissue [62]. In a study, which was injected as intraperitoneal at a dose 5 mg/kg of Paclitaxel diluted in 1 mL saline once a week for four weeks, it was reported that it caused increases in 8—OHdG and DNA damage according to the control group in rats, but administration of propolis, at a dose of 50 mg/kg dissolved in 1 mL distilled water orally once daily for four weeks, alleviated the toxic effect of Paclitaxel by diminishing oxidation state and DNA damage, preserving cell energy [63]. The content of the ration used in the study is given in Table 4. When examining the content of propolis in this study, it was observed that the content of flavonoids was high (Table 5 and Table 6). Due to that content, it was observed to have a strong antioxidant property and show that effect by preventing lipid peroxidation caused by CP toxicity.

Molecules such as calcium, ceramide and the Bcl-2 family, as well as proteins such as p53, caspase and cytochrome-c, and also mitochondrials, usually have an important role in the regulation of apoptosis. Whether or not a cell tends toward apoptosis depends on the heterodymus or homodymus form of the Bcl-2 family genes. The Bcl-2 family consists of proapoptotic and antiapoptotic groups. If proapoptotic proteins are greater in a cell, the cell tends toward apoptosis. If antiapoptotic proteins are greater, the cell tends toward apoptosis less [64,65]. While Bax is a proapoptotic member, Bcl-2 is an antiapoptotic member. In this study, these two important parameters were examined to determine apoptosis. It is known that CP induces apoptosis in CP-sensitive cells by activating Bax, leads to the release of cytochrome C into cytosol, and activates caspase. In addition, Bcl-2 protein regulated in CP-resistant cells has been revealed to be an important factor in CP resistance [66]. In the present study [66], it was found out that CP apparently induced apoptosis because of Bax protein increases in the liver and kidney tissues, whereas Bcl-2 protein decreased (Figure 1, Figure 2, Figure 4 and Figure 5), which is compatible with the literature. In the CP-related apoptosis; propolis and NP applications increased the release of Bcl-2 protein and decreased the release of Bax protein. In a study, in which galangin was used to prevent the renal tubular damage induced by CP in rats [67], it was determined that CP increased the expression of Bax, which is a proapoptotic protein, and decreased the expression of Bcl-2, which is an antiapoptotic protein. On the other hand, the use of galangin reversed that situation. According to the results of the present study, it was determined that apoptosis caused by CP in the liver and kidney tissues was reversed with the propolis and NP applications (Figure 1, Figure 2, Figure 4 and Figure 5). In addition, when examining the Bax/Bcl-2 rate, it was determined that there were significant differences between CP + NP-30 and propolis groups in terms of the liver and kidney tissues and the CP + NP-30 group gave effective results by significantly reducing the Bax level in tissues (Figure 3 and Figure 6). According to the results acquired, the fact that especially the second dose (30 mg/kg BW) of NP was much more effective than free propolis (100 mg/kg BW) at the cellular level was associated with the significant increase in propolis activity by nanotechnology, which thus made it possible to obtain better results despite using lower doses of propolis [54].

Oršolić et al. [68] administered propolis (50 mg/kg, ip) 7 and 3 days before inoculation of the Ehrlich ascites tumor (EAT) cells (2 × 10^6^, ip) in mice, and then applied the cisplatin (5 or 10 mg/kg, ip) 3 days after the inoculation of EAT cells at 37 °C and 43 °C. After the experimental period, they reported that the combination of propolis + cisplatin 5 mg/kg at 37 °C resulted in tumor growth inhibition and increased the survival of mice; propolis also reduced the toxic and genotoxic effect of cisplatin on normal cells without affecting the cytotoxicity of cisplatin on EAT cells. In another study, which was designed to investigate the in vitro anticancer effect of propolis ethanolic extract (PEE) and its protective role against methotrexate (MTX) toxicity in the Ehrlich acid carcinoma (EAC) experimental model, Salem et al. [69] reported that the PEE prompted cytotoxic effects in cancer cell lines and antitumor effects against the EAC mice model by reducing the tumor volume and count of viable tumor cells, with a significant increase in the life period of mice. Production of NP and its utilization in cancer cases are a new approach together with the progress of nanotechnology.

## 4. Materials and Methods

### 4.1. Drugs

Chitosan (low molecular weight, 50,000–190,000) and chemicals used for antioxidant analyses were supplied by commercial firms (Sigma-Aldrich, Istanbul, Turkey). Propolis was collected from Hatay Province, Turkey. Ethyl alcohol, acetic acid and Tween 80 chemicals used for NP synthesis were all purchased from firms (Merck, Istanbul, Turkey). Antibodies used for Western blot analyses were supplied by a firm (Abcam, Istanbul, Turkey). All chemicals were used in their received form without any further purification. The doses of CP and propolis have been determined according to Zheng et al. [70] and Seven et al. [71], respectively.

### 4.2. Animal Housing and Experimental Design

This study was conducted by following all procedures for the care and use of laboratory animals. The local Ethics Committee for Animal Experiments of Firat State University approved the study protocols (approval number: 2017/23-270). Fifty-six Sprague–Dawley male rats (6–8 weeks, 200–250 g) were obtained from the Laboratory Animal Research Center (Firat State University, Turkey). Fresh water and standard commercial pellet food (Table 4) were provided *ad-libitum*.

Rats were hosted in a controlled room at 22 ± 2 °C and 12 h dark/light cycle. The rats were divided into 7 experimental groups by balancing initial body weights (n = 8). (1) Control group: rats were injected with normal saline at a single dose (1 mL/kg, intraperitoneally). (2) Cisplatin group (CP): rats were administered CP intraperitoneally at a single dose of 3 mg/kg BW. (3) Propolis group: rats were administered propolis by gavage at a dose of 100 mg/kg BW per day for 21 days. (4) Alone NP group 10 (NP-10): rats were administered a dose of NP at 10 mg/kg BW per day by gavage for 21 days. (5) CP + Propolis group: rats were administered cisplatin at a single dose of 3 mg/kg BW intraperitoneally, and propolis at a dose of 100 mg/kg BW per day by gavage, for 21 days. (6) CP + NP-10: rats were administered cisplatin at a single dose of 3 mg/kg BW intraperitoneally, and NP at a dose of 10 mg/kg BW/day by gavage, for 21 days. (7) CP + NP-30: rats were administered cisplatin at single dose of 3 mg/kg BW intraperitoneally, and NP at a dose of 30 mg/kg BW per day by gavage, for 21 days.

### 4.3. Analysis of Propolis

#### 4.3.1. Extraction of Propolis

For the extraction of propolis, 100 mg sample (3 parallels) was extracted within 25 mL 60% ethanol, and incubated at room temperature for 6 days (vortexing every day). After the incubation period, the extract was sonicated in an ultrasonic bath (Ultrasonic Cleaner-VWR, Ankara, Turkey) for 10 min, and centrifuged at 4000 rpm (+4 °C, 10 min) (Andreas Hettich GmbH&Co.KG, Tuttlingen, Germany) [72].

#### 4.3.2. The Analysis of Total Phenolic Content

To determine the total phenolic content of propolis extracts, Folin–Ciocalteu phenol reagent (0.75 mL, 0.1 N) and Na_2_CO_3_ (0.75 mL, 6%) were added to 0.1 mL of each replicate. Gallic acid was used as a standard according to the method of Velioglu et al. [73]. One and half an hours later, the absorbance was spectrophotometrically measured at 725 nm (Table 5).

#### 4.3.3. Content of Total Flavonoid

Total flavonoid of extract was colorimetrically measured according to the method, which was expressed as quercetin equivalent, of Kim et al. [74]. A total of 1 mL of each extract was mixed with NaNO_2_ (0.3 mL, 5%) at t = 0 min. AlCl_3_ (0.3 mL, 10%) was added to the mix at t = 5 min. Six min later, NaOH (2 mL, 1 N) was added to it and then mixed. The absorbances were measured against the prepared water blank at 510 nm (Table 5).

#### 4.3.4. Total Antioxidant Capacity (TAC) of Propolis

The TAC was estimated by two different assays. The Cupric reducing antioxidant capacity (CUPRAC) and 2,2-diphenyl-1-picrylhydrazyl (DPPH) assays were performed according to Apak et al. [75] and Rai et al. [76], respectively. In all assays, Trolox was used as a standard.

##### Total Antioxidant Capacity According to the DPPH Method

The antioxidant activity of the propolis extract was evaluated on basis of the radical scavenging effect of the stable DPPH free radical [76]. For this aim, 0.1 mL of each extract was added 2 mL of DPPH (0.1 mM) in methanol solution. After the incubation at room temperature for 30 min in a test tube, the absorbances were spectrophotometrically measured against blank (methanol) at 517 nm (Table 5).

##### Total Antioxidant Capacity According to the CUPRAC Method

According to the CUPRAC method [75], 1 mL of CuCl_2_ (0.01M), 1 mL of neocuproine (0.0075 M) and 1 mL of NH_4_Ac buffer (pH 7.0) were mixed in a test tube. Then, 0.1 mL of extract was added to the mixture. Lastly, 1 mL of MQ water was added to the mixture to make the final volume 4.1 mL. After 1 h reaction time, the absorbances were measured at 450 nm (Table 5).

#### 4.3.5. Major Individual Phenolic Profile of Propolis

The extracts, which were filtered with a 0.45-µm membrane filter, were analyzed by the Waters W600 HPLC system coupled with PDA (Waters 996) detector [77]. The compound was separated with Luna^®^ 3 µm C18 100 Å, LC Column 150 × 4.6 mm, and Ea (Phenomenex, CA, USA). The mobile phase consisted of solvent A, Milli-Q water with 0.1% (*v*/*v*) trifluoroacetic acid (TFA) and solvent B, acetonitrile with 0.1% (*v*/*v*) TFA. A linear gradient was used as follows: 95% solvent A and 5% solvent B at 0 min, 65% solvent A and 35% solvent B at 45 min, 25% solvent A and 75% solvent B at 47 min and at 54 min returns initial conditions. The flow rate was 1 mL/min. Detection was done at 280, 312 and 360 nm. Identification was based on the retention times and characteristic UV spectra and quantification was done by external standard curves. All analyses were performed in triplicate (Table 6).

### 4.4. Preparation of Nano-Propolis

In this study, two different doses of eco-friendly NP (NP-10, 840 mg propolis/350 mL and NP-30, 1260 mg propolis/180 mL) were synthesized by the green sonication method at 25 °C (frequency 35 kHz, 320 W) [78,79,80].

#### 4.4.1. Nano-Propolis-10 (NP-10)

A total of 3.5 g chitosan was dissolved into 230 mL 2% *v*/*v* acetic acid solution in an ultrasonic bath for 15 min. Then, 1g of tween 80 was added into the solution and kept magnetic stirring (100 rpm) for 30 min under or at room temperature. A quantity of 840 mg of propolis (85%) was dissolved into 120 mL ethanol. Subsequently, the solution was sonicated at 50% amplitude for 10 min. Lastly, a homogeneous nanostructure was obtained and then stored at 4 °C for later use.

#### 4.4.2. Nano-Propolis-30 (NP-30)

A total of 1.8 g chitosan was dissolved into 120 mL 2% *v*/*v* acetic acid solution in an ultrasonic bath for 15 min. Then, tween 80 (1 g) was added into the solution above and kept magnetic stirring (100 rpm) under or at room temperature for 30 min. A quantity of 1260 mg of propolis (85%) was dissolved into 60 mL ethanol. Subsequently, the obtained solution was sonicated at 50% amplitude for 10 min. Finally, a homogeneous nanostructure was obtained and then stored at 4 °C for later use.

#### 4.4.3. Characterization Technique

Fourier Transform Infrared Spectrometer (FTIR) (Perkin Elmer Spectrum Two FTIR Spectrometer), (KBr powder, 4000 cm^−1^ to 500 cm^−1^ with a resolution of 4 cm^−1^ using 8 scans) and Scanning Electron Microscopy (SEM) (FE-SEM, JEOL 63335F) (gold coating, 20 kV accelerating voltage) were used to understand the morphological and chemical characterization of NP.

**Figure 7:**
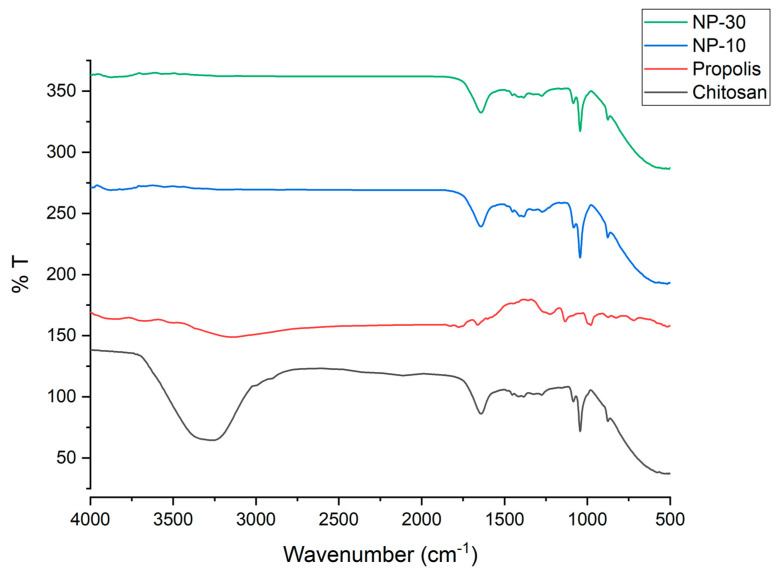
Nano-propolis-10 (NP-10; 840 mg/350 mL), nano-propolis-30 (NP-30; 1260 mg/180 mL), propolis and chitosan solutions (permanent phase) for FTIR data.

**Figure 8:**
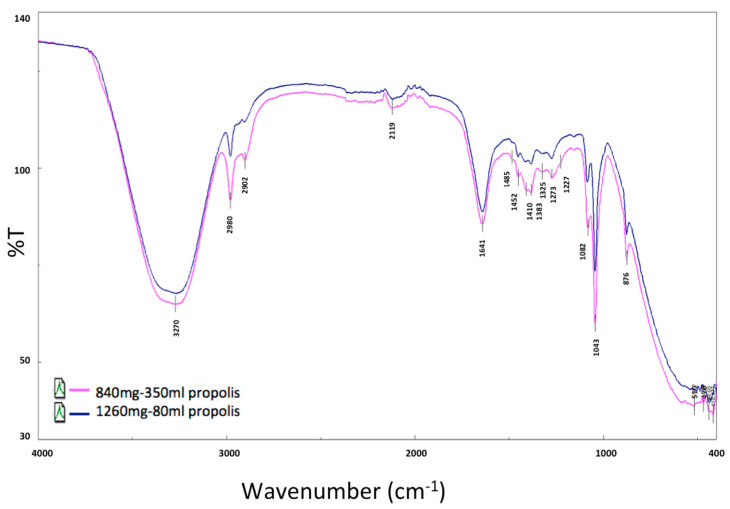
Nano-propolis-10 (NP-10; 840 mg/350 mL) and nano-propolis-30 (NP-30; 1260 mg/180 mL) for FTIR data.

The FTIR spectra of N-P-10, N-P-30 and solvent were shown in Figure 7 and Figure 8. The characteristic absorption band of the chitosan was at about 1560 cm^−1^, which was assigned to the stretching vibration of the amino group of chitosan. In addition, there was a band at about 1340 cm^−1^ assigned to the vibration of C–H. Another band near 3370 was due to amine NH symmetric vibration. The peak there of about 2927 cm^−1^ was a typical C–H vibration. The peaks around 900 and 1150 cm^−1^ were suited to the saccharide structure of chitosan. The wide peak near 1090 indicated C–O stretching vibration. Propolis extract had typical bands; at approximately 1165 cm^−1^, C–O and C–OH vibration; around 1435 cm^−1^, C–H vibration; near 1512, 1598, and 1630 cm^−1^, aromatic ring deformations; and around 1680 cm^−1^, C = O stretching of flavonoids and lipids. The spectrum of NP-10 and NP-30 showed characteristic bands both of chitosan (e.g., 1080 cm^−1^) and propolis extract (e.g., 1681, 1627, 1598, 1513 and 1434 cm^−1^), with slight and no significant shifts. In addition, at NP-10 and NP-30, the –OH band of chitosan showed a significant decrease because of chison–propolis bonding [81,82,83].

**Figure 9:**
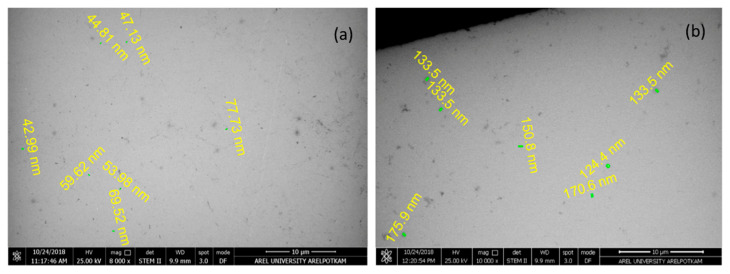
(**a**) Nano-propolis-10 (NP-10; 840 mg/350 mL) (**b**) Nano-propolis-30 (NP-30; 1260 mg/180 mL).

**Figure 10:**
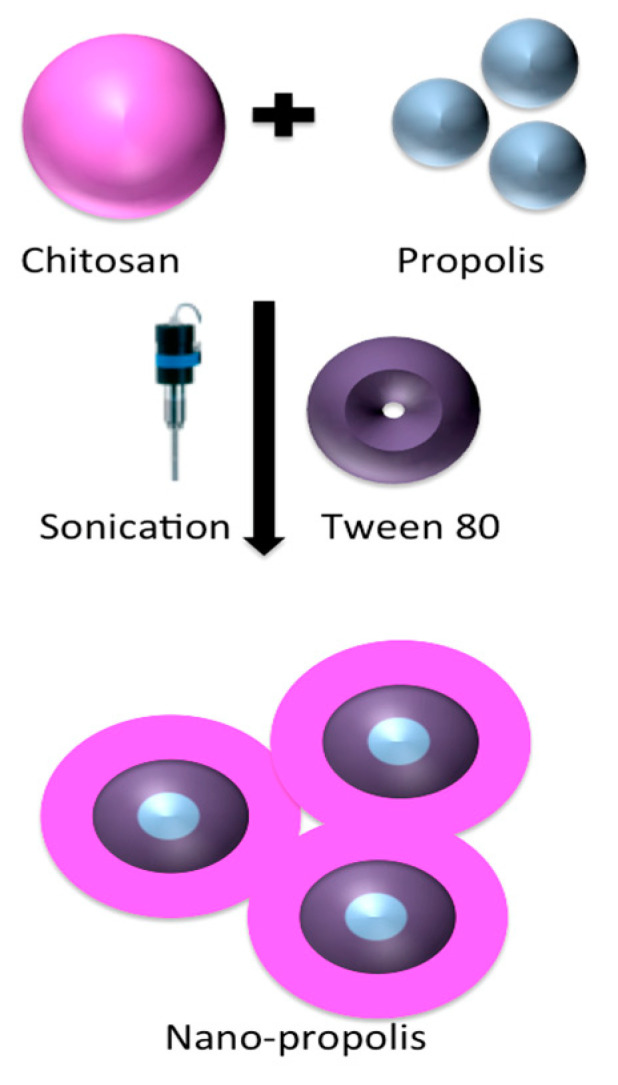
The schematic diagram of synthesis of nano-propolis.

SEM micrographs were used for morphological analysis of NP-10 and NP-30 nanoparticles (Figure 9). In both nanostructures, propolis interacted with chitosan as a spherical structure with a diameter of less than 200 nm and a narrow range. In addition, homogeneous distribution of propolis in the polymer matrix was observed. Experimental results showed that propolis was surrounded with polymer and interacted with the hydrophilic end (–OH group) of Tween 80 used as a stabilizing agent in nanosize in chitosan (-NH2) [84,85]. Although the homogeneity of the distribution did not change with increasing amounts of propolis, it was determined that the size of the particles increased. The interaction between chitosan and Tween 80 showed that NP were coated with chitosan (Figure 10).

### 4.5. Performance Parameters

Rats were housed in individual cages to determine the BW, BWC and FI at 1, 7, 14 and 21 days of the experiment. The FI, BW and BWC of groups were individually recorded (Table 1).

### 4.6. Blood Pparameters

Glucose, aspartate aminotransferase (AST), alanine transaminase (ALT), alkaline phosphatase (ALP), total protein, albumin, creatinine and blood urea nitrogen (BUN) were determined to the end of the study (Table 2).

### 4.7. Oxidative Stress Analyses

#### 4.7.1. Sample Collection and Homogenate Preparation

After the rats were decapitated, their tissues were washed with phosphate buffer and each tissue sample was wrapped in aluminum foil, placed in polyethylene bags and labeled. The tissues were kept −20 °C until analysis. The tissues were weighed and transferred to glass tubes, maintaining their coldness. Tris buffer (pH = 7.4) was added on the tissues in 1/10 ratio. Maintaining their coldness, the tissues were homogenized in the homogenizer. Total protein determination in tissue was analyzed using the Lowry method [86].

#### 4.7.2. Determination of Malondialdehyde (MDA) Levels

The amount of MDA produced in the tissues was used as an indicator of lipid peroxidation (LPO) level. MDA levels were measured according to the spectrophotometric method defined by Placer et al. [87]. The pink colored complex of MDA formed by thiobarbituric acid was measured spectrophotometrically at 532 nm (Table 3).

#### 4.7.3. Measurement of Catalase (CAT) Enzyme Level in Tissues

The CAT activities were determined using the method of Goth [88]. When the tissues were incubated with the substrate containing hydrogen peroxide (H_2_O_2_), hydrogen peroxide (H_2_O_2_) was cleaved to H_2_O and O_2_ by CAT activity. The ammonium molybdate added to the medium combined with H_2_O_2_ to terminate the reaction. During this period, the color change was measured spectrophotometrically at 405 nm (Table 3).

#### 4.7.4. Glutathione (GSH) Levels in Tissues

The GSH levels were measured spectrophotometrically at 412 nm according to the method of Sedlak and Lindsay [89]. The color intensity of the yellow colored complex formed by 5,5-dithio-bis (2-nitrobenzoic acid) (DTNB) was directly proportional to the concentration of GSH in the environment (Table 3).

#### 4.7.5. The Glutathione Peroxidase (GSH-Px) Activity

The GSH-Px activities were determined by the method of Lawrence and Burk [90]. The yellow color complex formed as a result of mixing the samples with DTNB solution at 412 nm on the spectrophotometer (Table 3).

### 4.8. Determination of Protein Expressions by Western Blotting Technique

Tissues were homogenized with cold RIPA lysis buffer, then centrifuged at 14,000 rpm at +4 °C and the supernatant was separated. Total protein contents were determined by the spectrophotometric method, which was defined by Smith et al. [91]. Samples were electrophoresed by loading polyacrylamide gel with equal amounts of protein (50 µg) in each well [92] and after sodium dodecyl sulfate—polyacrylamide gel electrophoresis (SDS-PAGE), specific proteins were transferred to polyvinylidene difluoride (PVDF) membrane by Western blotting [93]. The PVDF membranes were washed 3 times with TBS-T for 5 min to prevent nonspecific binding and then, blocked with 5% milk powder. Membranes were incubated overnight with appropriate primary (Bcl-2, Bax, and Beta actin) antibodies, and after incubation, the membranes were washed 3 times with TBS-T for 5 min. After incubation with the secondary antibodies for 1 h, the membranes were washed again with TBS-T for 5 min, followed by incubation, and the bands obtained using chemiluminescent conjugate were imaged on the chemiluminescence imaging system (Biorad ChemiDoc ™XRS +). The band densities in the obtained images were measured with the appropriate analysis system (Biorad Image Lab ™ Software version 5.2.1, Biorad Laboratories, Inc., USA). Protein expression levels were normalized to beta actin, which was used as internal control [94,95] (Figure 1, Figure 2, Figure 3, Figure 4, Figure 5 and Figure 6).

### 4.9. Statistical Analysis

In Western blott statistical analyses, target proteins were normalized to beta actin. Shapiro–Wilk normality analysis was used to determine whether the values obtained as a result of normalization were normally distributed or not. Shapiro–Wilk normality analysis showed normal distribution of the data. ANOVA one-way analysis of variance was used to compare group means. Differences between the groups were determined by Duncan test. IBM SPSS Statistics 22 package program was used for statistical analysis. The data are given as Mean ± SD. Significance is *p* < 0.05.

## 5. Conclusions

In the results, it was determined that the NP was more effective than propolis and, especially, the dose of 30 mg/kg BW was apparently effective in minimizing the liver and kidney damage caused by CP. Based on the study results, it is thought that the oral administration of (low dose of propolis) nanoparticles of propolis together with CP can make the treatment process more effective and more comfortable by reducing CP’s side effects (anorexia, weight loss, oxidative damage and apoptosis) in cancer patients. Therefore, in future research, NP should be examined in terms of drug interactions in the cancerous organism.

## Figures and Tables

**Table 1:** Effects of propolis and nano-propolis on body weight (BW—g/rat), body weight change (BWC—g/rat/day) and feed intake (FI—g/rat/day) of the experimental groups (Mean ± SD).

	Days	Control	CP	Propolis	NP-10	CP + Propolis	CP + NP-10	CP + NP-30	*p*
BW	IW	246.60 ± 3.94	247.75 ± 3.18	247.63 ± 13.42	245.33 ± 3.96	247.00 ± 1.48	243.93 ± 3.88	247.00 ± 3.63	NS
	7.	273.50 ± 8.75	260.44 ± 2.17	275.63 ± 13.09	270.67 ± 1.86	264.60 ± 4.56	260.36 ± 3.60	261.25 ± 3.32	NS
	14.	291.30 ± 3.52 ^a,b^	272.44 ± 3.63 ^c^	295.25 ± 3.40 ^a^	299.08 ± 2.96 ^a^	280.20 ± 9.29 ^b,c^	276.64 ± 2.97 ^c^	277.50 ± 4.32 ^b,c^	**
	21.	312.80 ± 1.69 ^a^	288.81 ± 2.38 ^b^	314.25 ± 13.21 ^a^	314.08 ± 4.67 ^a^	310.70 ± 10.26 ^a^	290.50 ± 3.90 ^b^	294.81 ± 6.42 ^a,b^	**
BWC	1–7	3.84 ± 1.10	1.81 ± 0.29	4.00 ± 0.50	3.62 ± 0.66	2.52 ± 0.68	2.35 ± 0.35	2.04 ± 0.50	NS
	8–14	2.54 ± 0.98	1.72 ± 0.40	2.80 ± 1.41	4.06 ± 0.62	2.23 ± 0.97	2.33 ± 0.50	2.32 ± 0.52	NS
	15–21	3.07 ± 0.52	2.34 ± 0.54	2.72 ± 1.49	2.14 ± 0.66	4.36 ± 1.17	1.98 ± 0.47	2.48 ± 0.76	NS
	1–21	3.15 ± 0.25 ^a,b^	1.95 ± 0.21 ^c^	3.18 ± 0.31 ^a,b^	3.28 ± 0.34 ^a^	3.03 ± 0.47 ^a,b^	2.22 ± 0.21 ^b,c^	2.28 ± 0.30 ^b,c^	**
FI	1–7	19.87 ± 0.86	19.59 ± 0.86	20.76 ± 0.47	19.89 ± 0.67	19.64 ± 1.08	19.33 ± 1.40	20.18 ± 0.91	NS
	8–14	22.80 ± 0.52	19.67 ± 1.23	19.30 ± 0.36	22.00 ± 1.00	19.52 ± 0.75	19.14 ± 0.89	20.89 ± 1.23	NS
	15–21	24.50 ± 0.49 ^a^	20.49 ± 0.32 ^c^	23.87 ± 0.11 ^a,b^	23.70 ± 1.11 ^a,b^	23.64 ± 1.35 ^a,b^	22.07 ± 0.46 ^b,c^	23.81 ± 0.75 ^a,b^	**
	1–21	22.39 ± 0.13 ^a^	19.92 ± 0.39 ^c^	21.31 ± 0.24 ^a,b,c^	21.87 ± 0.71 ^a,b^	20.93 ± 0.63 ^a,b,c^	20.18 ± 0.50 ^b,c^	21.63 ± 0.78 ^a,b,c^	*

IW: Initial weight; CP: cisplatin; NP-10: 10 mg/kg BW of nano-propolis; NP-30: 30 mg/kg body weight of nano-propolis; ^a,b,c^: Mean values with different superscripts within a row differ significantly; NS: non-significant; *: *p* < 0.05; **: *p* < 0.01.

**Table 2:** Effects of propolis and nano-propolis on biochemical parameters of experimental groups (Mean ± SD).

	Control	CP	Propolis	NP-10	CP + Propolis	CP + NP-10	CP + NP-30	*p*
GLU (mg/dL)	119.00 ± 1.58 ^d^	155.75 ± 4.33 ^a^	121.00 ± 4.23 ^d^	120.75 ± 0.85 ^d^	142.40 ± 1.17 ^b^	140.75 ± 2.10 ^bc^	130.40 ± 6.73 ^c,d^	***
AST (U/L)	224.60 ± 8.61 ^b^	298.80 ± 13.93 ^a^	223.67 ± 12.65 ^b^	222.60 ± 10.32 ^b^	242.40 ± 8.85 ^b^	238.83 ± 11.26 ^b^	234.14 ± 17.20 ^b^	**
ALT (U/L)	88.50 ± 3.74 ^b^	103.40 ± 4.26 ^a^	87.00 ± 5.19 ^b^	89.00 ± 1.78 ^a,b^	99.20 ± 9.23 ^a,b^	97.43 ± 4.60 ^a,b^	93.88 ± 2.01 ^a,b^	*
ALP (U/L)	289.75 ± 4.84 ^c^	355.40 ± 8.15 ^a^	282.50 ± 2.32 ^c^	285.75 ± 2.06 ^c^	312.20 ± 4.05 ^b^	313.75 ± 4.59 ^b^	311.50 ± 7.16 ^b^	***
TP (g/dL)	6.13 ± 0.05 ^a,b^	5.57 ± 0.10 ^c^	6.10 ± 0.12 ^a,b^	6.26 ± 0.07 ^a^	5.78 ± 0.14 ^b,c^	5.86 ± 0.18 ^a,b,c^	5.88 ± 0.13 ^a,b,c^	*
Alb (g/dL)	3.62 ± 0.06 ^a,b^	3.27 ± 0.09 ^c^	3.63 ± 0.06 ^a,b^	3.75 ± 0.06 ^a^	3.48 ± 0.03 ^b^	3.52 ± 0.04 ^b^	3.54 ± 0.04 ^b^	***
Cre (mg/dL)	0.28 ± 0.01 ^b,c^	0.33 ± 0.01 ^a^	0.28 ± 0.02 ^b,c^	0.25 ± 0.02 ^c^	0.31 ± 0.01 ^a,b^	0.31 ± 0.02 ^a,b^	0.30 ± 0.01 ^a,b^	*
BUN (mg/dL)	52.80 ± 1.85 ^b,c^	65.00 ± 0.53 ^a^	49.75 ± 1.38 ^c,d^	47.25 ± 0.63 ^d^	56.40 ± 0.93 ^b^	54.33 ± 1.45 ^b^	54.00 ± 1.29 ^b^	***

CP: cisplatin; NP-10: 10 mg/kg body weight of nano-propolis; NP-30: 30 mg/kg body weight of nano-propolis; Glu: glucose; AST: aspartate aminotransferase; ALT: alanine transaminase; ALP: alkaline phosphatase; TP: total protein; Alb: albumin; Cre: creatinine; BUN: blood urea nitrogen; ^a,b,c,d^: Mean values with different superscripts within a row differ significantly; *: *p* < 0.05; **: *p* < 0.01; ***: *p* < 0.001.

**Table 3:** Effects of propolis and nano-propolis on MDA (nmol/g), GSH (nmol/g), GSH-Px (IU/g protein), and CAT (kU/g protein) values in the liver and kidney tissues of the experimental groups (Mean ± SD).

		Control	CP	Propolis	NP-10	CP + Propolis	CP + NP-10	CP + NP-30	*p*
**Liver**	MDA	13.10 ± 0.77 ^c^	17.32 ± 0.74 ^a^	13.28 ± 0.77 ^b,c^	12.97 ± 0.49 ^c^	15.10 ± 0.41 ^b^	14.07 ± 0.67 ^b,c^	12.30 ± 0.24 ^c^	***
	GSH	2.23 ± 0.07 ^b,c^	1.80 ± 0.06 ^d^	2.21 ± 0.08 ^b,c^	2.32 ± 0.08 ^a,b^	2.06 ± 0.07 ^c^	2.21 ± 0.08 ^b,c^	2.55 ± 0.08 ^a^	***
	GSH-Px	21.44 ± 1.84 ^b^	14.60 ± 0.69 ^c^	22.97 ± 1.25 ^a,b^	22.25 ± 1.57 ^a,b^	19.79 ± 2.47 ^b^	23.56 ± 1.01 ^a,b^	26.42 ± 1.22 ^a^	***
	CAT	2.80 ± 0.16 ^a^	1.13 ± 0.05 ^d^	2.68 ± 0.27 ^a^	2.80 ± 0.15 ^a^	1.63 ± 0.04 ^c^	2.01 ± 0.08 ^b,c^	2.52 ± 0.17 ^a,b^	***
**Kidney**	MDA	21.32 ± 0.97 ^c,d^	33.53 ± 1.07 ^a^	20.41 ± 0.73 ^d^	20.76 ± 1.34 ^c,d^	27.82 ± 2.10 ^b^	24.07 ± 0.89 ^c^	20.73 ± 0.63 ^c,d^	***
	GSH	1.93 ± 0.08 ^b^	1.56 ± 0.08 ^c^	1.90 ± 0.05 ^b^	1.94 ± 0.08 ^b^	1.95 ± 0.06 ^a,b^	1.99 ± 0.09 ^a,b^	2.21 ± 0.06 ^a^	***
	GSH-Px	30.43 ± 1.66 ^a,b^	17.78 ± 1.34 ^d^	26.46 ± 1.36 ^b,c^	27.01 ± 2.64 ^b,c^	23.00 ± 1.45 ^c^	28.99 ± 1.29 ^a,b^	33.86 ± 1.62 ^a^	***
	CAT	1.79 ± 0.17 ^b^	1.00 ± 0.12 ^d^	1.86 ± 0.20 ^b^	2.16 ± 0.12 ^a,b^	1.40 ± 0.12 ^c^	1.78 ± 0.06 ^b^	2.27 ± 0.07 ^a^	***

CP: cisplatin; NP-10: 10 mg/kg body weight of nano-propolis; NP-30: 30 mg/kg body weight of nano-propolis; MDA: malondialdehyde; GSH: glutathione; GSH-Px: glutathione peroxidase; CAT: catalase; ^a,b,c,d^: Mean values with different superscripts within a row differ significantly; ***: *p* < 0.001.

**Table 4:** Nutrient composition of diet used in the study.

Nutritional Composition	%	Nutritional Composition	%
Dry matter ^1^	92.5	Ether extract ^1^	3.21
Crude ash ^1^	6.48	Ca ^2^	0.89
Crude protein ^1^	24.00	P ^2^	0.98
Crude cellulose ^1^	6.15	Metabolizable energy (kcal/kg) ^2^	2650

^1^—analyzed; ^2^—calculated.

**Table 5:** The total phenolic and flavonoid content and antioxidant capacity of propolis for three parallel (Mean ± SD).

Content	mg/g
Total phenolic content, GAE	17.18 ± 0.45
Total flavonoid content, QE	42.28 ± 1.23
Total antioxidant capacity—CUPRAC, TEAC	143.16 ± 1.31
Total antioxidant capacity—DPPH, TEAC	20.09 ± 1.31

GAE: Gallic acid equivalent; QE: Quercetin equivalent; CUPRAC: Cupric reducing antioxidant capacity; TEAC: Trolox equivalent antioxidant capacity; DPPH: 2,2-diphenyl-1-picrylhydrazyl.

**Table 6:** Major individual phenolic substances and quantities defined in propolis for three parallel (Mean ± SD).

Phenolics	mg/g	Phenolics	mg/g
Caffeic acid	0.17 ± 0.00	Pinostrobin	2.93 ± 0.03
Vanillin	0.23 ± 0.00	Pinocembrin	1.22 ± 0.15
Ferulic acid	0.36 ± 0.00	Chrysin	2.94 ± 0.07
t-cinnamic acid	3.95 ± 0.00	Galangin	0.09 ± 0.01
Pinobanksin	0.58 ± 0.00		
